# Coix seed oil ameliorates cancer cachexia by counteracting muscle loss and fat lipolysis

**DOI:** 10.1186/s12906-019-2684-4

**Published:** 2019-10-15

**Authors:** Huiquan Liu, Lu Li, Jun Zou, Ting Zhou, Bangyan Wang, Huihui Sun, Shiying Yu

**Affiliations:** Cancer Center, Tongji Hospital, Tongji Medical College, Huazhong University of Science and Technology, No. 1095, Jiefang Street, Wuhan, 430030 People’s Republic of China

**Keywords:** Coix seed oil, Cancer cachexia, Muscle loss, Lipolysis, AMPK, NF-κB

## Abstract

**Background:**

Cancer cachexia is a cancer-induced multifactorial debilitating syndrome directly accounting for 20% of cancer deaths without effective therapeutic approaches. It is extremely urgent to explore effective anti-cachexia drugs to ameliorate muscle and fat loss in cachexia patients.

**Methods:**

Lewis lung carcinoma bearing C57BL/6 mice were applied as the animal model to examine the therapeutic effect of Coix seed oil (CSO) on cancer cachexia. The food intake and body weight change were monitored every 3 days throughout the experiment. The IL-6 and TNF-α levels in serum were detected by ELISA assay. Several key proteins involved in muscle wasting and fat lipolysis were tested by Western blot to identify the potential mechanism of CSO.

**Results:**

Administration of CSO through gavage significantly prevented body weight loss and ameliorated systemic inflammation without affecting food intake and tumor size. The weight and histological morphology of gastrocnemius muscle and epididymal adipose tissue in CSO-treated mice were also improved. In mechanism, we found that CSO decreased the expression of MuRF1 and the ratio of phospho-p65 (Ser536) to p65 in muscle tissue. Meanwhile, cancer-induced activation of HSL and AMPK was also inhibited by CSO administration.

**Conclusion:**

Coix seed oil exerts an anti-cachexia pharmaceutical effect by counteracting muscle and adipose tissue loss most likely through regulating NF-κB-MuRF1 and AMPK-HSL pathway.

## Background

Cancer cachexia is a devastating multifactorial disorder characterized by clinical symptoms of fatigue, anorexia and ongoing body weight (BW) loss [1]. It occurs in more than half of cancer patients, leading to reduced therapeutic efficacy, worse treatment tolerance, poor quality of life and prognosis [2, 3]. In fact, the incidence of cancer cachexia is uniformly very high at the end of patients’ life regardless of cancer type [4], directly resulting in more than 20% of cancer deaths [5]. Though much progress in exploring the pathogenesis of cancer cachexia has been achieved in the past decades, there are still few approaches to alleviate cachexia in clinical practice, let alone to reverse it.

Skeletal muscle wasting is the most prominent feature of cachexia in cancer patients. Previous studies have shown that the E3 ubiquitin ligase MuRF-1 which belongs to the ubiquitin-proteasome system and its upstream nuclear transcription factor kappaB (NF-κB) signaling pathway play vital roles in the process of muscle wasting. Activation of NF-κB caused by tumor-derived substances or systemic inflammation stimulates MuRF-1 expression resulting in proteolysis in muscle tissue [6, 7]. Importantly, a wild range of drugs targeting the NF-κB pathway such as baicalin [8] and carboxyamidotriazole [9] have been proved to attenuate cancer cachexia in tumor-bearing mice. Excessive loss of fat mass, another pathologic change usually accompanied with muscle loss, has been proposed to aggravate the cancer cachexia condition. The increased free fatty acids in blood circulation caused by lipolysis could excessively accumulate in muscle tissue resulting in several biological changes, such as the activation of ubiquitin lipases in muscle cells [10]. Besides, lipolysis and adipose tissue wasting were reported to probably occur before muscle loss [11, 12]. The pivotal functional proteins involved in lipolysis are adipose triglyceride lipase (ATGL) and hormone sensitive triglyceride lipase (HSL), which are regulated by multiple pathways, for example, AMP-activated protein kinase (AMPK) signaling [13–15].

Considering the multifactorial pathogenesis and complicated multiorgan dysfunction in cancer cachexia, developing a targeted medical compound that indeed alleviates cachexia is truly difficult. Till now, there is no standard chemical drug clinically approved for cancer cachexia. Anamorelin, a selective novel ghrelin receptor agonist stimulating appetite, is the most promising drug officially approbated for cancer cachexia [16, 17]. However, we believe that ameliorating cachexia needs not only to improve appetite but also to counteract muscle and adipose tissue loss.

Coix seed extract using standardized pharmaceutical-grade technology is an oily substance which has been formulated into an emulsion named Kanglaite Injection® (KLT) for clinical use. Recently, a randomized, open-label, phase III clinical study revealed that KLT plus gemcitabine could significantly improve progression-free survival of pancreatic cancer patients compared to gemcitabine alone [18]. Meanwhile, it was reported KLT treatment significantly improved quality of life evaluated by the Functional Assessment of Anorexia Cachexia Therapy Score [18]. Besides, Wu and colleagues reported that Kanglaite treating at a dose of 200 ml/day for 21 days significantly increased average body weight of stage IV lung cancer patients by 55.9% compared with that before treatment [19].

Given that Kanglaite was reported to inhibit NF-κB pathway [20], in the current study, we tried to explore whether Coix seed oil (CSO) had a therapeutic effect on cancer cachexia and to explain the underlying mechanism using generally accepted animal model. Both muscle and adipose tissue loss were involved in.

## Methods

### Cell culture and CSO preparation

Lewis lung carcinoma (LLC) cells were provided by the Department of Pathogenic Biology at Tongji Medical College (Huazhong University of Science and Technology, Wuhan, China) and were cultured at 37 °C with 5% CO_2_ using Dulbecco’s modified Eagle’s medium containing 10% fetal bovine serum (Gibco; Thermo Fisher Scientific, Waltham, MA, USA) and 1% penicillin/streptomycin (Boster Biological Technology, Wuhan, China). Before tumor inoculation, LLC cells were centrifuged at 1000 rpm at 4 °C for 5 min and resuspended in phosphate buffer saline (PBS). CSO was prepared by the Zhejiang Kanglaite Pharmaceutical Co.Ltd. using seeds harvested from specified fields in the Zhejiang Province of China through high performance liquid chromatography (HPLC) technology as previously published [20]. There are plenty of active ingredients such as hexadecenoic acid, octadecoic acid (C18), octacenic acid (C18–1), octadedienoic acid (C18–2) and so on [21] mixed in this yellow oily liquid.

### Animal studies

Specific pathogen-free C57BL/6 mice aged 5–6 weeks were purchased from laboratory animal center, Huazhong University of Science and Technology. All mice were housed free for chow and water in the condition of stable temperature and humidity with a regular 12-h light-dark cycle under the specific pathogen-free environment. C57BL/6 mice were randomly assigned into three groups (7 mice per group): 1) control mice (Ctrl group) injected subcutaneously with PBS in the right flank of axilla; 2) cachectic mice (CA group) injected subcutaneously with 1 × 10^6^ LLC cells in the right flank of axilla receiving daily gavage of 2.5 ml/kg sterile water; 3) cachectic mice treated with daily gavage of 2.5 ml/kg Coix seed oil (CA + Coix group). The dosage of administration in the present study was derived from that published previously [22] and was also the converted dose equivalent to human use on the basis of the body surface area [23]. The body weight of each mouse and cumulative food intake (combined of all seven mice in each group) were monitored every 3 days following tumor implantation. Twenty-four days after the tumor implantation, animals were anesthetized with a 1.2% avertin solution (0.5 g 2,2,2-tribromoethanol powder dissolved into 1 ml 2-methyl-2-butanol and 39 ml PBS) at a dose of 0.16 ml/10 g BW intraperitoneally and sacrificed via exsanguination. Epididymal adipose tissue, bilateral gastrocnemius muscle, and subcutaneous tumor mass were integrally harvested and weighted. We randomly selected 5 pieces of epididymal adipose tissue and 5 pieces of the gastrocnemius muscle in each group for subsequent hematoxylin-eosin staining (HE staining). The other pieces of mice tissues were immediately transferred to − 80 °C ultra-low temperature freezer. All experimental protocols were approved by the Institutional Animal Care and Use Committee at Tongji Medical College, Huazhong University of Science and Technology and were in accordance with the National Institutes of Health Guide for the Care and Use of Laboratory Animals.

### Enzyme-linked Immunosorbent assay (ELISA)

After mice were anesthetized, blood samples were collected through the retro-orbital sinus and centrifuged at 2500 rpm at 4 °C for 15 min within 1 h to get serum for ELISA test. The quantitation of IL-6 and TNF-α in serum was performed using IL-6 and TNF-α ELISA Kit (Bio-swamp, Wuhan, China) according to the manufacturer’s protocol.

### HE staining

The epididymal adipose tissue and gastrocnemius muscle of mice were fixed in 4% paraformaldehyde, embedded in paraffin, sectioned transversely and stained with hematoxylin and eosin solutions. Morphological images were acquired using an optical microscope (Sunny Optical Technology, China).

### Western blot analysis

Tissues stored at − 80 °C were ground in liquid nitrogen and cleaved at 4 °C in RIPA lysis buffer with 1% PMSF and 1% phosphorylase inhibitor cocktail (Beyotime Technology, Shanghai, China) for 30 min. The protein concentration was measured using bicinchoninic acid (BCA) assay (Beyotime Biotechnology) and protein samples were heated for 5 min at 100 °C with SDS-PAGE sample buffer (Boster Biological Technology, Wuhan, China). Next, 30μg total protein samples of each group were separated through electrophoresis in 10% SDS-PAGE. Subsequently, proteins were transferred onto polyvinylidene difluoride (PVDF) membranes and blocked in 5% bovine serum albumin at room temperature for 1 h. Then the proteins on PVDF membranes were incubated overnight at 4 °C with primary antibodies against NF-κB p65 (p65; dilution 1:1000; cat# 8242; Cell Signaling Technology, Danvers, MA, USA), phosphorylated NF-κB p65 (phospho-p65; dilution 1:1000; cat# 3033, Ser536; Cell Signaling Technology), MuRF1 (dilution 1:1000; cat# 55456–1-AP; Proteintech Technology, Wuhan, China), GAPDH (dilution 1:2000; cat# BA2913; Boster Biological Technology, Wuhan, China), HSL (dilution 1:1000; cat# 4107; Cell Signaling Technology), phosphorylated HSL (dilution 1:1000; cat# 4126, Ser660; Cell Signaling Technology), AMPK (dilution 1:1000; cat# 2532; Cell Signaling Technology), phosphorylated AMPK (dilution 1:1000; cat# 2535, Thr172; Cell Signaling Technology), and beta-actin (dilution 1:1000; cat# sc-70,319; Santacruz Biotechnology, Dallas, Texas, USA). After being washed by Tris-buffered saline with 0.1% Tween-20 (TBST) for 5 times, the membranes were then incubated with the corresponding secondary antibody conjugated with horseradish peroxidase (dilution 1:10000, Aspen Biotechnology, Wuhan, China). Lastly, following TBST washed for 3 times, membranes were exposed using ECL solution (Servicebio Technology, Wuhan, China). Quantification of the protein bands was performed using Image J software. Protein expression levels were normalized to GAPDH or β-actin expression.

### Statistical analysis

SPSS software version 19.0 (IBM SPSS, Armonk, NY, USA) was used for statistical analysis and the data were exhibited as mean ± standard error of the mean (mean ± SEM). For difference analysis between two groups, we used the student’s t-test. For three groups, one-way ANOVA and post-hoc test were performed. A *p*-value of less than 0.05 was considered statistically significant.

## Results

### CSO prevented body weight loss of LLC bearing mice

**Fig. 1:**
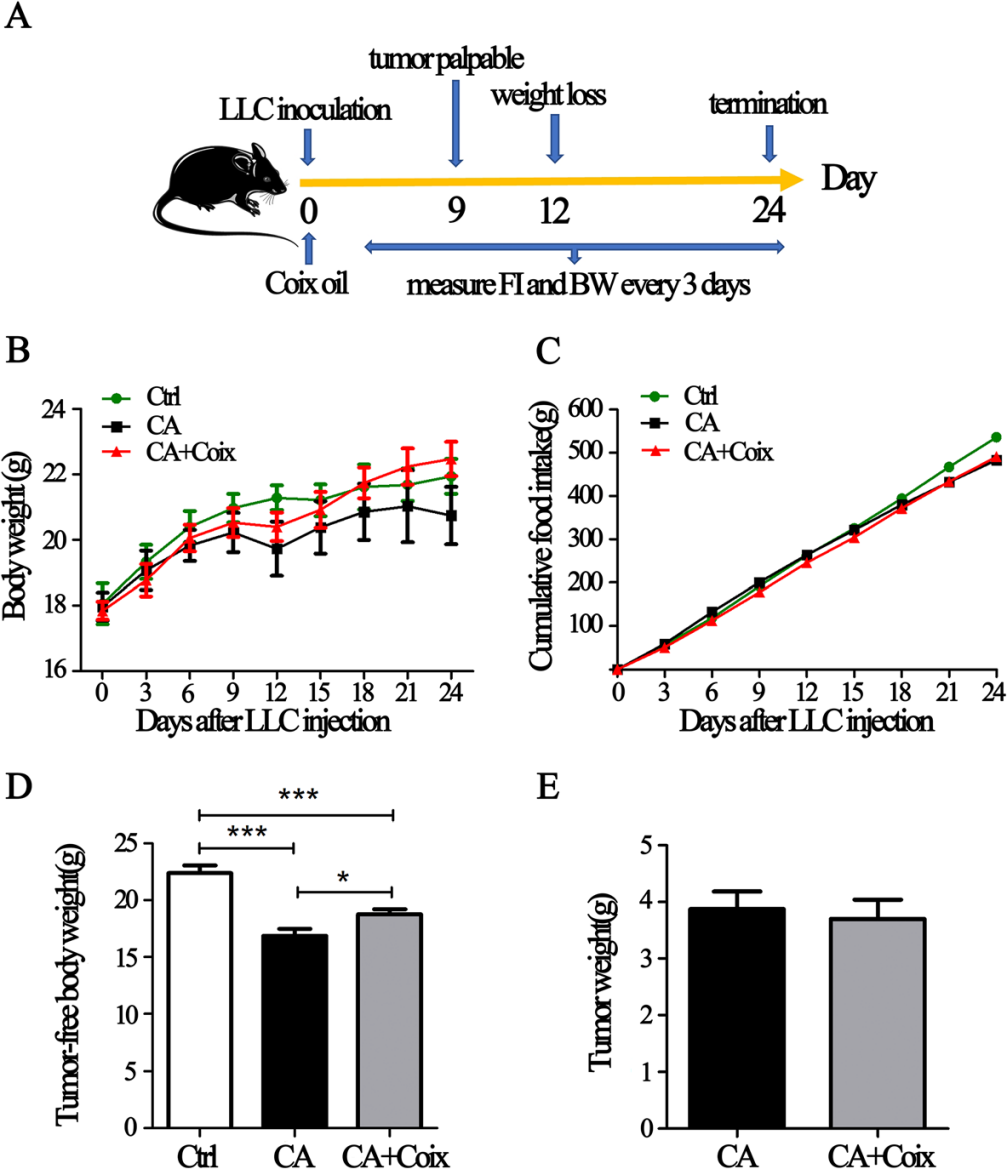
CSO prevented body weight loss of LLC-bearing mice. **a**. The whole work flow of the animal experiment. **b**.The average body weights of the mice in three groups (Ctrl, CA, CA + Coix) were measured every 3 days. **c**. Changes of cumulative food intake combined of all seven mice in each group were recorded over the whole experiment. **d, e**.The tumor-free body weight (D) and tumor weight (E) were measured after mice were sacrificed. Values were expressed as mean ± SEM (*P<0.05, ***P<0.001)

As shown in Fig. 1a and Fig. 1b, subcutaneous tumor masses were palpable 9 days after LLC inoculation. BW loss occurred on day 12 in the CA group, while the BW of control mice continuously increased. During day 12–21, on account of the rapid growth of tumor mass, the BW of the CA group increased gradually once again. On day 24, we detected BW loss in CA group for the second time, which may be ascribed to severely cancer-induced cachexia. A previous study has shown that on day 24, the tumor-free body mass decreased for almost 15% compared to control mice indicating the existence of cachectic condition [24], which is consistent with our results (Fig. 1d).

Six hours after LLC inoculation, the mice in CA + Coix group were administrated through gavage for 2.5 ml/kg CSO per day per mouse. This group exhibited nearly on-going weight increase during the whole course (Fig. 1a). Importantly, on day 24 the average BW of CA + Coix group did not decline as obviously as that of CA group. The tumor-free body weight between these two groups showed a significant difference (Fig. 1d). These results demonstrated that CSO administration attenuated BW loss of LLC bearing mice.

Anorexia is a common accompanying symptom of cachexia clinically. Thus, we also monitored the food intake change during the developing process of cachexia. However, even on day 24, there was no obvious difference in the average food intake between the Ctrl, CA and CA + Coix group (data not shown). Cumulative food intake line in Fig. 1c delineated a decreased total energy intake of tumor-bearing mice compared to Ctrl, whereas no difference between CA and CA + Coix group existed. These results showed that the anti-cachexia effects of CSO are not through stimulating the appetite.

Previous studies have elucidated KLT injection has an anti-cancer effect. However, although our data showed that after CSO administration tumor mass decreased slightly, there was no statistical difference in tumor mass between the CA and CA + Coix group (Fig. 1e).

### CSO alleviated systemic inflammation, counteracted muscle and fat loss

**Fig. 2:**
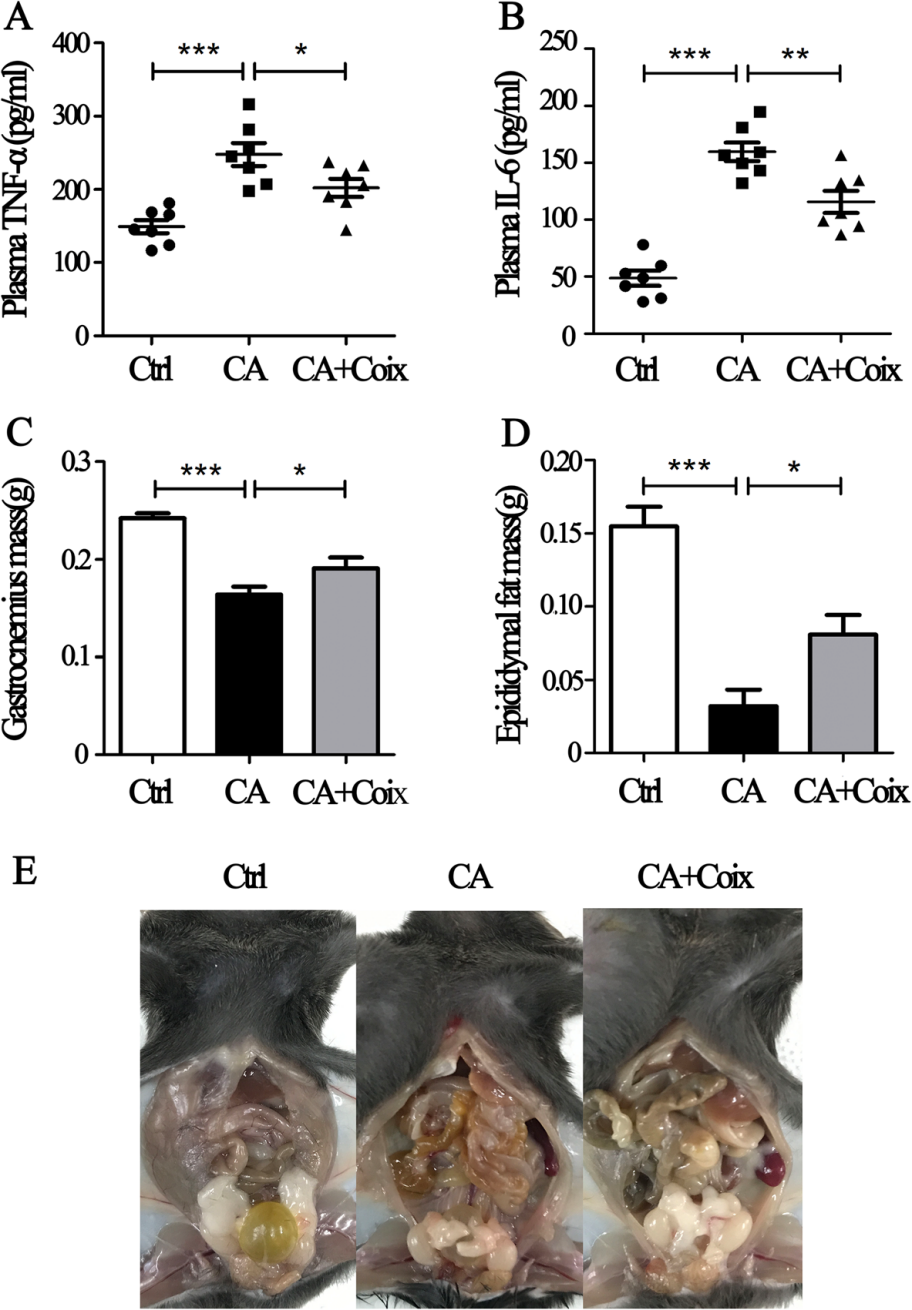
CSO alleviated systemic inflammation, counteracted muscle and fat loss. A, B. CSO administration significantly reduced the levels of TNF-α (**a**) and IL-6 (**b**) in the serum of cachectic mice. **c**. Tumor-bearing mice suffered from significant muscle loss while CSO significantly ameliorated the muscle wasting. **d**. CSO administration remarkably elevated epididymal fat weight in cachectic mice. **e**. Representative figures of epididymal fat inthree groups (Ctrl, CA, CA + Coix). Values were expressed as mean ± SEM (*P<0.05, **P<0.01, ***P<0.001)

In addition to BW, the other salient features of cancer cachexia are systemic inflammation, muscle loss with or without fat loss. Recently, an exosome-related study demonstrated that LLC-derived Hsp70 and Hsp90 are responsible for the elevation of circulating cytokines in tumor-bearing mice, which can act alone or in combination to aggravate muscle and fat loss [8, 25]. Our study confirmed the increased levels of TNF-α and IL-6 in the CA group and found that CSO administration significantly reduced the levels of both TNF-α and IL-6 in serum, indicating the relief of systemic inflammation (Fig. 2a and Fig. 2b).

Muscle loss is the most prominent feature which mainly accounts for BW loss. We completely peeled off bilateral gastrocnemius muscle and measured them precisely. Results showed that tumor-bearing mice suffered from significant muscle loss while CSO obviously ameliorated muscle wasting (Fig. 2c). Although adipose tissue loss is not requisite for cachexia diagnosis, in the animal model of LLC-bearing mice, fat loss usually occurs before muscles loss and the elevated free fatty in muscles tissue may aggravate muscle wasting. Therefore, preventing fat loss is another important aspect to relieve cachexia. On day 24, we detected extremely obvious epididymal adipose tissue loss (Fig. 2e) and the epididymal adipose tissue in several tumor-bearing mice even disappeared. As shown in Fig. 2d, the CSO administration remarkably elevated epididymal fat weight compared to the CA group. These results demonstrated that CSO not only counteracted muscle loss but also neutralized adipose tissue wasting.

### CSO attenuated muscle fiber atrophy and adipose tissue wasting induced by cancer cachexia

**Fig. 3:**
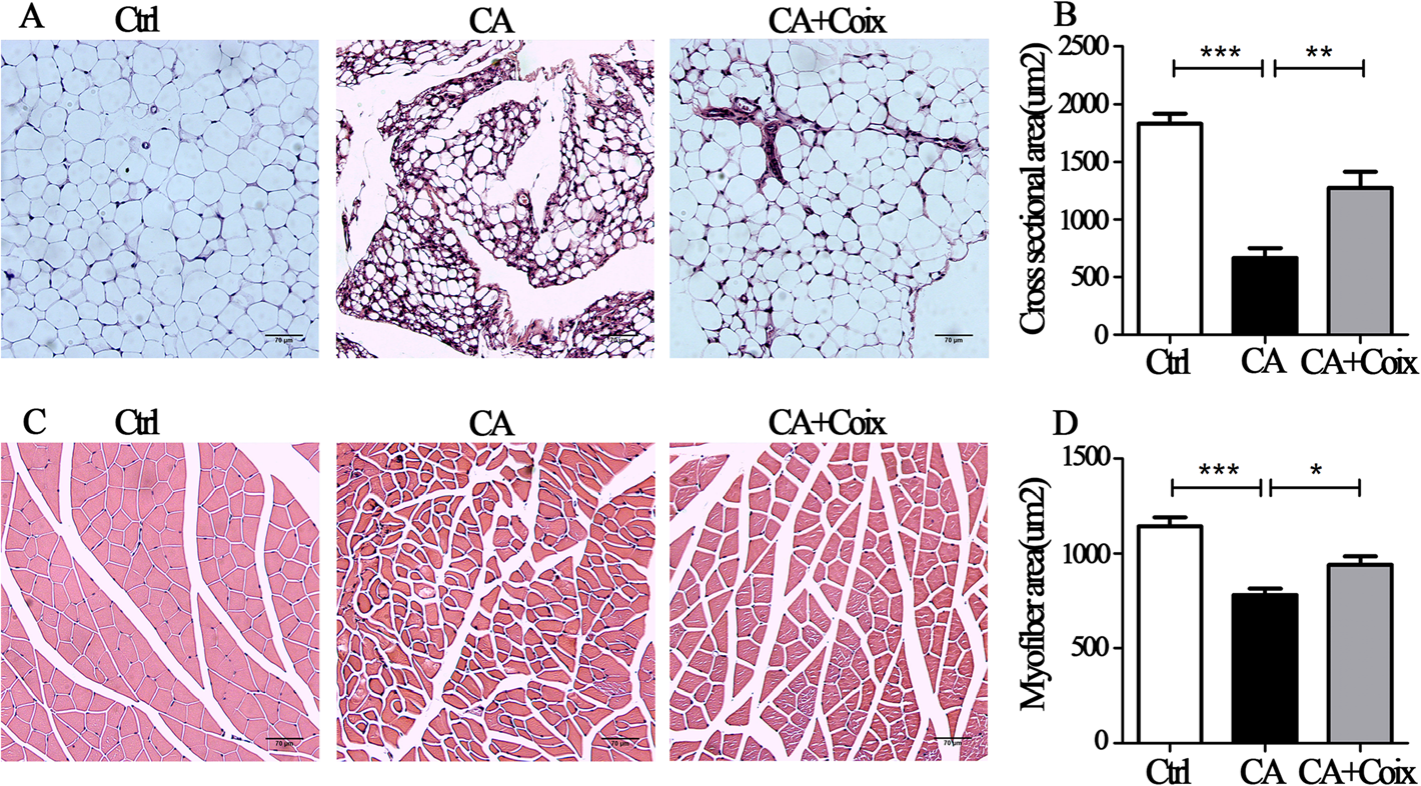
CSO attenuated muscle fiber atrophy and adipose tissue wasting induced by cancer cachexia. **a**. Representative figures of HE staining of epididymal adipose tissue in three groups (Ctrl, CA, CA + Coix). **b**.The quantitative comparison of cross-sectional area (um^2^) of adipocytes between three groups (Ctrl, CA, CA + Coix) revealed that CSO administration counteracted adipose tissue wasting. **c**. Representative cross-sectional histological staining of muscle fiber in three groups (Ctrl, CA, CA + Coix). **d**.The quantification of myofiber cross-sectional area showed that CSO administration attenuated muscle fiber atrophy. Values were expressed as mean ± SEM (*P<0.05, **P<0.01, ***P<0.001)

The anti-cachexia effect of CSO was further confirmed histologically in adipose and muscle tissue. Fig. 3a exhibits representative HE staining of adipocytes in epididymal fat, which shows conspicuous morphological differences between the three groups. When the cross-sectional area was qualified by Image J, we found that adipose tissue in tumor-bearing mice severely shrank and CSO gavage predominantly interdicted fat wasting (Fig. 3b). As for muscle tissue, the CA group displayed overall sparse histological characteristics: the spaces between muscle fibers seem wider and the fiber thinner compared to those in the control and CA + Coix group (Fig. 3c). There was a significant difference in myofiber area between the CA and Ctrl group while the CA + Coix group exhibited a statistically increase in mean fiber area compared to CA group (Fig. 3d). Briefly, CSO attenuated cancer cachexia in LLC bearing mice by reducing both fat and muscle wasting.

### The NF-κB and AMPK pathway were involved in anti-cachexia effects of CSO

**Fig. 4:**
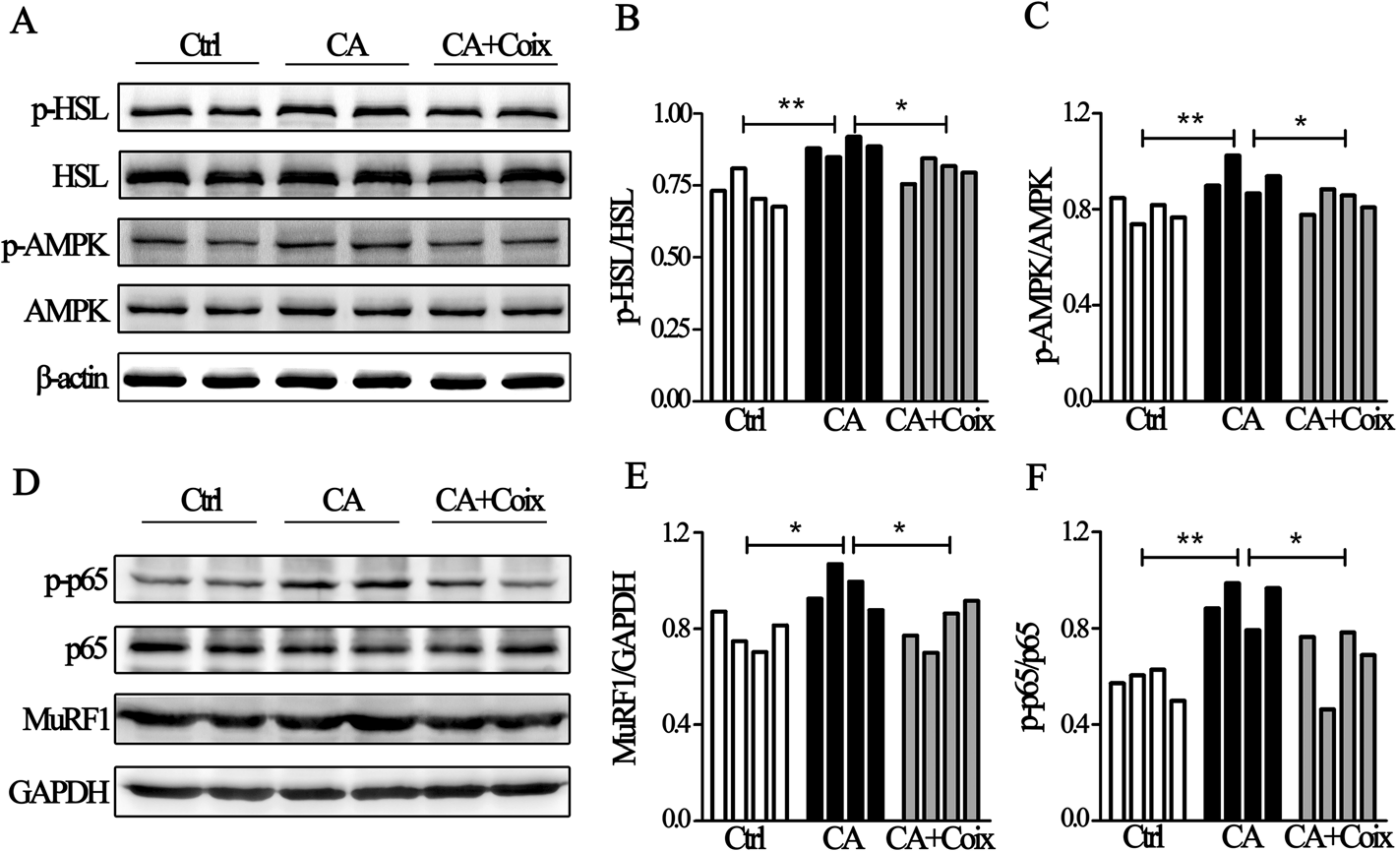
The NF-κB and AMPK pathway were involved in anti-cachectic effects of CSO. **a**. Representative Western-blot images exhibit the expression of p-HSL (Ser660), HSL, p-AMPK (Thr172), AMPK inthe Ctrl group, CA group, and CA + Coix group. **b**.The band densityanalysis showed that p-HSL/HSL in adipose tissue differed significantly between three groups (4 samples per group). **c**.The quantification of band density showed that p-AMPK/AMPK differed significantly between three groups (4 samples per group). **d**.Representative Western-blot images exhibit the expression of phospho-p65 (Ser536), p65 and MuRF1 inthe Ctrl group, CA group, and CA + Coix group. **e**.The quantification of band density revealed the increased ratio of MuRF1 to GAPDH in muscle tissue induced by cachexia was decreased by CSO administration (4 samples per group). **f**. The band densityanalysis exhibited that phospho-p65/p65 was elevated in CA group compared to Ctrl group while CSO administration significantly decreased the ratio (4 samples per group). Values were expressed as mean ± SEM (*P<0.05, **P<0.01)

Next, we wanted to explore the potential pharmacological mechanism of CSO in counteracting muscle and fat loss. Lipolysis is one of the most common physiological processes of fat wasting and phospho-HSL exerts critical function in lipolysis. Through western blot we observed that the value of phospho-HSL / HSL significantly raised in the CA group compared to Ctrl, suggesting lipolysis was involved in fat loss of cancer cachexia. As shown in Fig. 4a and Fig. 4b, CSO notably down-regulated the ratio of phospho-HSL (Ser660) to HSL, in other words, inhibited lipolysis in adipose tissue. In deep to discover the underlying mechanism, we evaluated whether AMPK signaling, one of the upstream pathways regulating HSL phosphorylation and energy balance, took part in this lipolysis. It was shown that cancer cachexia induced an increased ratio of phospho-AMPK (Thr172) to AMPK while CSO significantly restrained the phosphorylation of AMPK in epididymal adipose tissue (Fig. 4a, c). Accordingly, CSO counteracted adipose loss most likely through targeting AMPK-HSL signaling pathway.

As has been noted, systemic inflammation is one of the most salient features of cancer cachexia and NF-κB represents the main signaling pathway within cells response to cytokines. Cumulated studies have shown that serum cytokines such as IL-6 and TNF-α are elevated in cachexia mice and NF-κB played a vital role in muscle wasting. Given that in our study TNF-α and IL-6 level were decreased in serum by CSO administration, we tried to examine whether the NF-κB pathway was influenced by CSO. Results revealed that in LLC bearing mice, MuRF1 level was increased in cachectic muscle tissue while CSO decreased it to a certain degree (Fig. 4d, e), which indicated CSO ameliorated muscle protein degeneration. Elevated phospho-p65 and p65 are reliable indicators for NF-κB pathway activation, therefore we simultaneously detected these two proteins. Band analyses suggested that the increased phospho-p65 (Ser536) / p65 ratio caused by cancer was restrained by CSO (Fig. 4d, f). As MuRF1 is a downstream protein of NF-κB pathway, we shall extrapolate that CSO alleviates muscle wasting through inhibiting NF-κB- MuRF1 pathway.

## Discussion

Cancer cachexia is a devastating cancer-associated deteriorating syndrome characterized by muscle loss with or without fat loss [26]. The maintenance of skeletal muscle mass is important to ensure the quality of life and potentially prolongs the survival time of cancer patients. However, till now there are no therapeutic approaches ameliorating muscle and fat loss in clinical practice. It is extremely urgent to explore effective anti-cachexia drug to alleviate symptoms of cachexia patients. In this study, we demonstrated that: (1) CSO attenuated BW loss, muscle and adipose tissue loss, and systemic inflammation in LLC bearing mice; (2) CSO counteracted muscle fiber atrophy by targeting NF-κB pathway; (3) CSO ameliorated adipose tissue wasting by inhibiting HSL and AMPK activation.

As previously reported, the emulsion of coix seed oil called Kanglaite Injection® (KLT) exerted antineoplastic effect both in vivo and in vitro. Yuan and colleges illustrated that KLT exhibited pronounced anti-tumor and immunostimulatory activities in LLC bearing C57BL/6 mice by regulating NF-κB signaling pathway [27]. Intraperitoneal injection of KLT into nude nice inoculated with HepG2 cells had also been proved to significantly inhibit tumor growth [28]. In vitro, KLT was demonstrated to inhibit the epithelial-mesenchymal transition of colorectal cancer cells through suppressing NF-κΒ activation [29]. Furthermore, the synergistic anti-tumor effect of KLT with chemotherapeutic drugs has been observed in clinical practice [30] and clarified in mechanism [31]. What makes this especially exciting is that recently a clinical study reported patients with advanced lung carcinoma receiving standard KLT injection for 21 days showed a 55.9% increase in BW [19]. In this study, we found that CSO attenuated muscle loss and adipose tissue wasting in cachectic mice without affecting food intake and tumor burden, which laid the groundwork for CSO to be clinically used in cachectic cancer patients.

However, the anti-tumor activity of CSO was not observed in LLC-bearing C57BL/6 mice. This discrepancy may be due to the method of drug administration. Here we used pure CSO without excipient to treat the cachectic mice through gavage. Recently, Qu reported that oral multicomponent microemulsions containing CSO and ginsenoside Rh2 exerted synergistic antitumor effect and shrank xenograft of drug-resistant breast tumor [32]. Orally administrated CSO in that form was demonstrated to be able to accumulate at the tumor site after crossing the intestines as intact vehicles into the blood circulation, which were not interrupted by gut microbiota. We speculated the anti-tumor effects of this oil without excipient most likely been disturbed by gut microbiota which has been identified to influence chemotherapeutic efficacy [33]. Besides, we believe that emulsified CSO is a functional anti-cancer drug through systematically regulation in vivo. For the tumor inoculated subcutaneously which not proliferated in the original location, CSO probably could not maximize its antineoplastic effect sufficiently. Meanwhile, considering our data showed an anti-neoplastic trend of CSO without statistically significance, the discrepancy may also be attributed to the limited sample size.

Systemic inflammation is regarded as the crucial alteration that induces muscle atrophy in cancer cachexia [34, 35]. There exists considerable evidence suggesting TNF-a and IL-6 are the most relevant cytokines inducing muscle wasting and promoting the development of cachexia [36–38]. It has been commonly accepted that elevated serum cytokines convey biological effects on tissue cells via the NF-κB pathway [6, 39, 40]. The NF-κB p65 is normally sequestered within the cytoplasm. When it was activated by phosphorylation, cytoplasmic to nuclear localization of NF-κB p65 will take place to regulate transcription of effector genes [41]. In muscle cells, one of the downstream targeted genes of NF-κB pathway is MuRF-1, which plays a central role in muscle protein degeneration [42]. Here we indicated CSO could reduce the tumor-induced elevation of MuRF1 in muscle tissue through inhibiting phosphorylation of the NF-κB p65 at the Ser536 site, which also coincides with previous studies revealing the anti-tumor mechanism of KLT [20, 43].

The AMP-activated protein kinase has been proposed as a key protein for lipid metabolism to promote fatty acid oxidation in various tissues. Recently, its potential role in regulating adipose lipolysis was characterized by promoting phosphorylation of two key steatolysis-associated protein, ATGL and HSL [44–46]. In the present study, we validated CSO decreased activation of HSL and AMPK in epididymal adipose tissue. Similar to our research, Miao [47] reported that Pyrrolidine Dithiocarbamate could not only inhibit muscle atrophy but also regulate energy metabolism via AMPK-HSL pathway. However, due to extremely low solubility of CSO in water, we did not perform in vitro experiments evaluating biological effects and mechanism of CSO in C2C12 cells or 3 T3-L1 adipocytes. Although our results showed that cachectic symptoms were relieved and NF-κB along with AMPK-HSL pathway were influenced by CSO administration, without backward verification using exogenous pathway activator, we still cannot sufficiently conclude the anti-cachexia effects of CSO are mediated by NF-κB and AMPK-HSL pathway.

## Conclusion

Collectively, our study revealed the exciting anti-cachexia effect and potential mechanism of CSO in counteracting muscle and adipose tissue loss, which may shed light on the clinical use of CSO or KLT to alleviate cancer cachexia and improve quality of life in cancer patients.

## Data Availability

The datasets used and/or analyzed in this study are available from the corresponding author on reasonable request.

## Abbreviations

AMPK: Adenosine 5′-monophosphate activated protein kinase
ATGL: Adipose triglyceride lipase
BW: Body weight
CSO: Coix seed oil
ELISA: Enzyme-Linked Immunosorbent Assay
HE staining: Hematoxylin-eosin staining
HSL: Hormone sensitive triglyceride lipase
IL-6: Interleukin-6
KLT: Kanglaite Injection®
LLC: Lewis lung carcinoma
MuRF1: Muscle ring finger-1
NF-κB: Nuclear factor-κB
PBS: Phosphate buffer saline
TNF-α: Tumor necrosis Factor-α
